# Supplementary material for: Collective cell migration and metastases induced by an epithelial-to-mesenchymal transition in Drosophila intestinal tumors
Source: Nat Commun. 2019 May 24;10:2311. doi: 10.1038/s41467-019-10269-y (PMC6534551; doi:10.1038/s41467-019-10269-y)
Supplement: Supplementary file 1 — Supplementary Information [file 41467_2019_10269_MOESM1_ESM.pdf]

Supplementary Information

Collective cell migration and metastases induced by an epithelial-to-mesenchymal transition in *Drosophila* intestinal tumors

Campbell et al

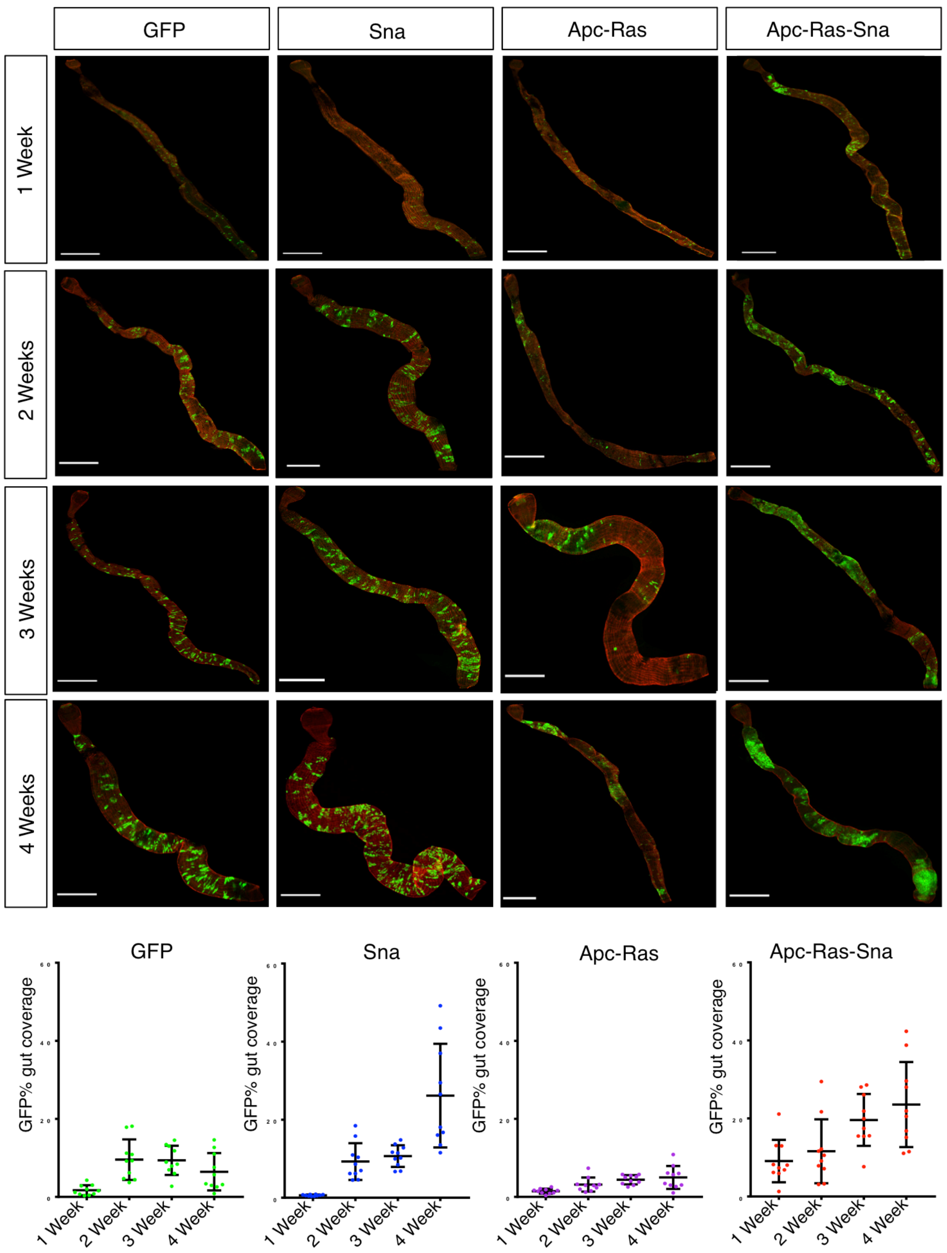

Supplementary Figure 1: Tumor burden in the midgut of flies bearing Apc-Ras clones increases over time. Midguts dissected from flies bearing GFP clones, Sna clones, Apc-Ras clones or Apc-Ras-Sna clones (GFP, green; Phalloidin, red) at 1 week, 2 weeks, 3 weeks and 4 weeks. Midgut clones in Apc-Ras and Apc-Ras-Sna increase in size over time, generating tumor-like overgrowths between 2-4 weeks after clone induction. GFP% whole-gut coverage was quantified per condition at 1 week, 2 weeks, 3 weeks and 4 weeks. n = 10 guts per time point. Error bars display standard deviation around the mean. Scale bars = 1000μm.

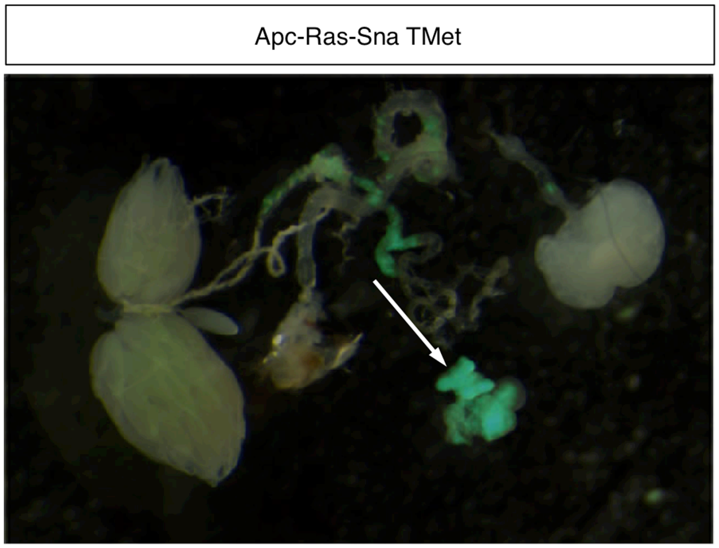

Supplementary Figure 2: At T1 stage a piece of a dissected TMet (arrow) is implanted into the abdomen of a wild type host female fly.

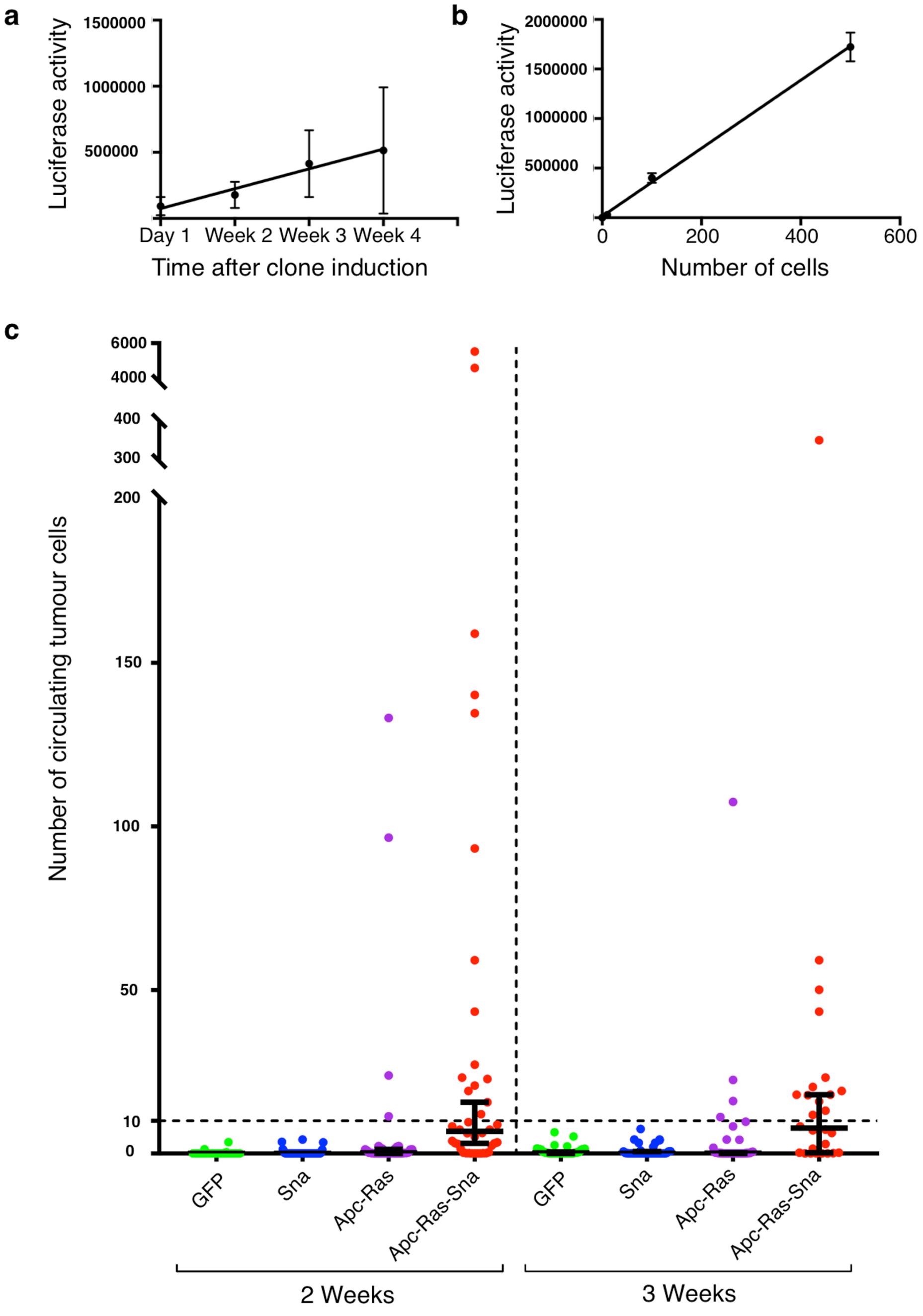

Supplementary Figure 3: Luciferase activity can be used to assay for the presence of tumor cells with a sensitivity of down to 10 cells.

(**a**) Lysates were prepared from adult flies and tested for luciferase activity. Error bars represent standard deviation (**b**) *Drosophila* midguts were dissected from Apc-Ras-Sna flies four weeks after induction. In order to obtain single cell suspensions, samples were incubated in PBS containing 0,4mg/ml dispase (Gibco) for 15 min at 37ºC, syringed

using a 27G needle and washed twice with PBS. After selecting for the viable population (propidium iodide negative), GFP^+ cells were isolated by fluorescence activated cell sorting (FACS) using a FACSAria 2.0 (BD Biosciences). GFP^+ cells were sorted into aliquots of 10, 100 and 500 cells and tested for luciferase activity. This experiment was done in triplicate Error bars represent standard deviation. A linear correlation (r=0.9994, p=0.0006) was observed between the number of cells isolated and the amount of luciferase activity detected. (**c**) The number of CTC's found in haemolymph extracted from individual flies from each condition at 2 weeks and 3 weeks after clone induction. At 2 weeks n = 34, 46, 36 and 40 for GFP, Sna, Apc-Ras and Apc-Ras-Sna respectively. At 3 weeks n = 40, 34, 38 and 28 for GFP, Sna, Apc-Ras and Apc-Ras-Sna respectively. The threshold of cells considered significant (10) is displayed by a dashed-line. Error bars display 95% confidence intervals around the median value.

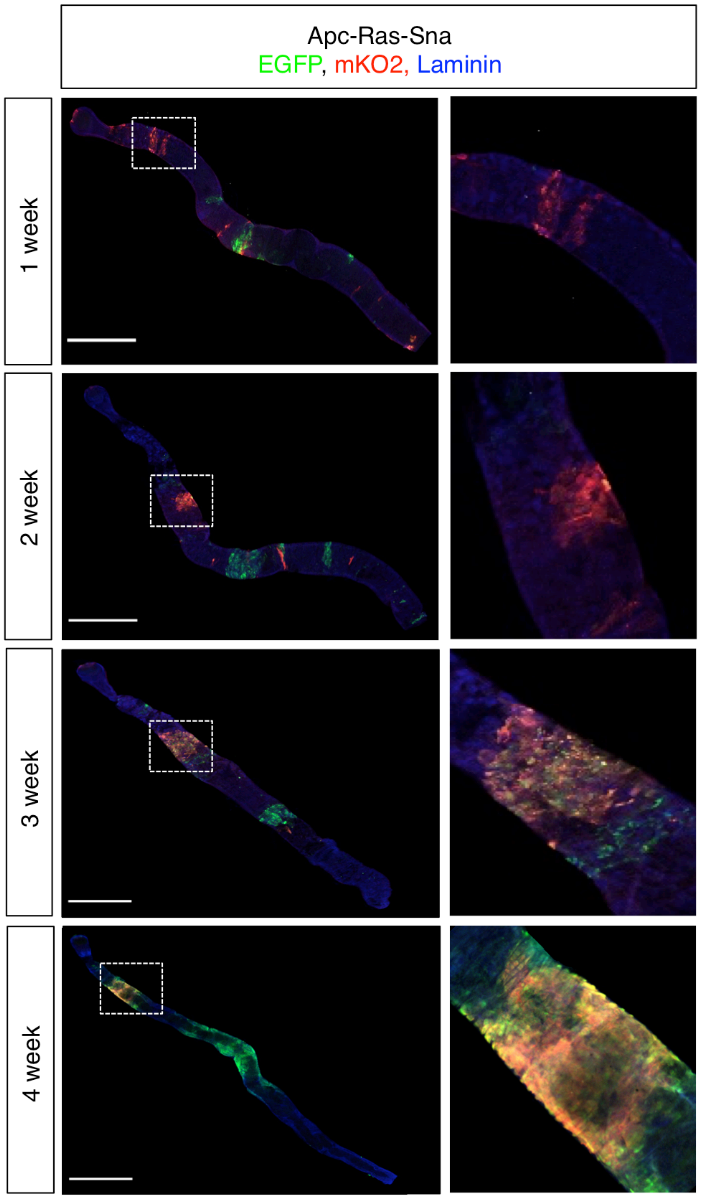

Supplementary Figure 4: The dBrainbow reporter enables the tracing of clone behaviour over time. Midguts dissected from flies bearing Apc-Ras-Sna clones and the dBrainbow reporter. Fixed midguts were stained for GFP (visualises EGFP, green), RFP (visualises mKO2, red) and Laminin (blue) and shown at 1 week, 2 weeks, 3 weeks and 4 weeks. The dashed box (left column) depicts the areas enlarged on the right. Scale bars = 1000µm.
